# Predicting Major Adverse Cardiovascular Events After Cardiac Surgery Using Combined Clinical, Laboratory, and Echocardiographic Parameters: A Machine Learning Approach

**DOI:** 10.3390/medicina61081323

**Published:** 2025-07-23

**Authors:** Mladjan Golubovic, Velimir Peric, Marija Stosic, Vladimir Stojiljkovic, Sasa Zivic, Aleksandar Kamenov, Dragan Milic, Vesna Dinic, Dalibor Stojanovic, Milan Lazarevic

**Affiliations:** 1 Clinic of Cardiovascular Surgery, University Clinical Center Nis, 18000 Nis, Serbia; velperic@gmail.com (V.P.); marija91nis@gmail.com (M.S.); drdraganmilic@gmail.com (D.M.); dalibor.stojanovic08@gmail.com (D.S.); 2 Medical School of Nis, University of Nis, 18000 Nis, Serbia; kamenovcs@gmail.com; 3 Institute for Treatment and Rehabilitation Niska Banja, 18205 Niska Banja, Serbia; serbvlada@yahoo.com (V.S.); dr_m.lazarevic@hotmail.com (M.L.); 4 Clinic for Cardiac Surgery, University Clinical Center Nis, 18000 Nis, Serbia; zivicdr@gmail.com; 5 Clinic for Anesthesiology, University Clinical Center Nis, 18000 Nis, Serbia; vesnadinic1981@gmail.com

**Keywords:** major adverse cardiovascular events, coronary artery bypass, aortic valve replacement, predictive modeling, machine learning

## Abstract

*Background and Objectives:* Despite significant advances in surgical techniques and perioperative care, major adverse cardiovascular events (MACE) remain a leading cause of postoperative morbidity and mortality in patients undergoing coronary artery bypass grafting and/or aortic valve replacement. Accurate preoperative risk stratification is essential yet often limited by models that overlook atrial mechanics and underutilized biomarkers. *Materials and Methods:* This study aimed to develop an interpretable machine learning model for predicting perioperative MACE by integrating clinical, biochemical, and echocardiographic features, with a particular focus on novel physiological markers. A retrospective cohort of 131 patients was analyzed. An Extreme Gradient Boosting (XGBoost) classifier was trained on a comprehensive feature set, and SHapley Additive exPlanations (SHAPs) were used to quantify each variable’s contribution to model predictions. *Results:* In a stratified 80:20 train–test split, the model initially achieved an AUC of 1.00. Acknowledging the potential for overfitting in small datasets, additional validation was performed using 10 independent random splits and 5-fold cross-validation. These analyses yielded an average AUC of 0.846 ± 0.092 and an F1-score of 0.807 ± 0.096, supporting the model’s stability and generalizability. The most influential predictors included total atrial conduction time, mitral and tricuspid annular orifice areas, and high-density lipoprotein (HDL) cholesterol. These variables, spanning electrophysiological, structural, and metabolic domains, significantly enhanced discriminative performance, even in patients with preserved left ventricular function. The model’s transparency provides clinically intuitive insights into individual risk profiles, emphasizing the significance of non-traditional parameters in perioperative assessments. *Conclusions*: This study demonstrates the feasibility and potential clinical value of combining advanced echocardiographic, biochemical, and machine learning tools for individualized cardiovascular risk prediction. While promising, these findings require prospective validation in larger, multicenter cohorts before being integrated into routine clinical decision-making.

## 1. Introduction

Major adverse cardiovascular events (MACE) remain a significant source of postoperative morbidity and mortality in patients undergoing coronary artery bypass grafting (CABG) and/or aortic valve replacement (AVR) [1,2,3]. Despite continuous advances in surgical techniques and perioperative care, these procedures are still associated with substantial cardiovascular risk. MACE typically include cardiovascular death, myocardial infarction (MI), stroke, and other life-threatening postoperative complications such as low cardiac output syndrome (LCOS), arrhythmias, and acute kidney injury (AKI) [2,4]. A growing body of evidence has emphasized the need to refine risk prediction tools beyond conventional scoring systems such as the EuroSCORE and the Society of Thoracic Surgeons’ (STS) risk calculator. These traditional models often fall short in capturing the complex interplay of functional, biochemical, and structural cardiac parameters that drive adverse outcomes in cardiac surgery patients [5,6].

Recent studies have increasingly highlighted the prognostic relevance of atrial function and atrioventricular geometric remodeling as emerging markers of cardiovascular risk. Among these, total atrial conduction time (TACT), a Doppler-derived echocardiographic parameter, serves as a robust and noninvasive surrogate for atrial electromechanical delay. Prolongation of TACT has been shown to reflect interatrial conduction disturbances, increased atrial fibrosis, and electrical remodeling, all of which are established substrates for atrial fibrillation and related hemodynamic instability in the perioperative setting. Beyond its association with arrhythmogenesis, prolonged TACT has been independently linked to higher rates of postoperative complications and prolonged hospital stay, especially in patients undergoing cardiac surgery [7,8,9].

In parallel, valvular annular morphology—particularly the tricuspid and mitral annular orifice areas—has emerged as a key determinant of atrial and ventricular interaction. The mitral annular orifice area reflects left atrial–ventricular coupling, and its enlargement may signal chronic volume overload or impaired diastolic compliance, which can compromise cardiac efficiency and promote remodeling. On the other hand, the tricuspid annular orifice area acts as a sensitive marker of right heart burden. Even in the absence of overt tricuspid regurgitation, tricuspid annular dilatation has been shown to predict poor outcomes, particularly in surgical cohorts, by indicating subclinical right atrial dysfunction and elevated pulmonary pressures [10,11,12]. These structural parameters are not only reflective of chronic hemodynamic stress but may also represent modifiable surgical targets. Recent evidence suggests that their integration into preoperative risk stratification could provide incremental prognostic value over traditional indices such as ejection fraction or comorbidity scores, especially in identifying patients at risk of MACE despite preserved global ventricular function. The clinical utility of these measures lies in their ability to capture early maladaptive changes, both electrical and geometric, that precede overt clinical deterioration [11,12].

In addition to structural and functional cardiac parameters, biochemical markers such as high-density lipoprotein (HDL) cholesterol have shown prognostic utility in cardiovascular surgery. HDL is known for its anti-inflammatory, antioxidant, and endothelial-protective effects, and lower preoperative HDL levels have been associated with an increased risk of adverse cardiac events. However, HDL has not been extensively evaluated in the context of surgical MACE risk prediction, representing a potentially underappreciated variable [13,14].

Given the multifactorial nature of MACE and the potential non-linear interactions among predictors, traditional regression models may be insufficient to capture the complexity of perioperative cardiovascular risk. In contrast, machine learning approaches such as gradient boosting can accommodate high-dimensional data and detect subtle, non-additive patterns, offering improved predictive performance and flexibility [6,15,16,17,18,19]. Therefore, the aim of this study was to develop and validate a predictive model for MACE in patients undergoing CABG and/or AVR using both conventional logistic regression and an explainable machine learning approach. Our methodology integrates clinical, biochemical, and echocardiographic variables, including underexplored physiological parameters such as total atrial conduction time and annular valve geometry. By applying SHAPs [6,15,16], we aimed to ensure model transparency and facilitate clinical interpretability. This study addresses a critical gap in perioperative risk stratification by combining predictive performance with explainable outputs relevant to surgical decision-making.

## 2. Materials and Methods

### 2.1. Study Design and Patient Selection

This research was designed as a prospective, observational, single-center study that enrolled a total of 131 patients undergoing elective or urgent cardiac surgery, specifically isolated CABG, isolated AVR with a mechanical prosthesis, or combined procedures. The study was conducted over a five-month period beginning in May 2024 at the Clinic for Cardiac Surgery, University Clinical Center in Niš, Serbia. The study adhered to the ethical principles outlined in the Declaration of Helsinki and was approved by both the Ethics Committee of the Faculty of Medicine, University of Niš, and the Ethics Committee of the University Clinical Center in Niš.

Patients undergoing mitral or tricuspid valve surgery were excluded due to the markedly higher incidence of postoperative atrial fibrillation in this subgroup, as well as the limited number of such procedures during the study period. The rationale for focusing exclusively on CABG and AVR was also based on the frequent coexistence of aortic stenosis and coronary artery disease, which share overlapping risk factors and exhibit similar pathophysiological mechanisms that influence postoperative outcomes.

The primary outcome of interest was the occurrence of MACE during the postoperative in-hospital period. MACE were defined as a composite outcome including cardiovascular death, myocardial infarction, stroke, and postoperative atrial fibrillation (POAF). While POAF is not typically included in classical MACE definitions, it was incorporated here due to its well-established clinical relevance in the perioperative setting, where it is associated with prolonged ICU and hospital stay, an increased risk of thromboembolism, and long-term cardiovascular morbidity. This extended definition reflects emerging perspectives in cardiac surgery risk modeling but may reduce comparability with studies using more restrictive endpoints. These events were systematically assessed based on standardized diagnostic criteria and clinical documentation, ensuring consistency in endpoint adjudication.

All patients underwent intensive postoperative monitoring for a minimum of seven days. Monitoring included continuous electrocardiographic surveillance, daily evaluation of 12-lead ECGs, and structured clinical assessments conducted by the attending cardiac surgical and anesthesiology teams. The preoperative evaluation consisted of a comprehensive medical history, physical examination, standard laboratory testing, including markers of myocardial injury, the lipid profile, renal function, chest radiography, transthoracic echocardiography, and electrocardiography. Anesthetic risk was assessed using the American Society of Anesthesiologists (ASA) classification, while the severity of heart failure symptoms was graded according to the New York Heart Association (NYHA) functional classification.

### 2.2. Variables and Data Collection

All variables used in this study were collected prospectively and categorized into three main domains: clinical, biochemical, and echocardiographic. Preoperative data were collected within 48 h prior to the surgical procedure, following standardized protocols.

Clinical variables included demographic characteristics such as age and gender, along with significant comorbidities including diabetes mellitus (DM), arterial hypertension (HTA), a history of myocardial infarction (MI), chronic obstructive pulmonary disease (COPD), and peripheral vascular disease (PVD). In addition, intraoperative use of milrinone was recorded as a binary variable, and the occurrence of POAF was noted as part of the early postoperative course.

Biochemical parameters were derived from routine preoperative laboratory panels and included hemoglobin concentration, erythrocyte count, serum creatinine, fibrinogen, cardiac troponin I (cTnI), and high-density lipoprotein cholesterol (HDL). All analyses were performed in the institutional central laboratory using certified standard operating procedures.

Echocardiographic measurements were obtained using transthoracic echocardiography (TTE) performed with a GE Vivid E95 system (GE HealthCare, Chicago, IL, USA). Parameters assessed included left ventricular ejection fraction (EF), TACT, the mitral annular orifice area, the tricuspid annular orifice area, minimal early diastolic filling velocity (EKK min), the right atrial area, and right atrial ejection fraction (RAEF). All echocardiographic assessments were conducted by board-certified cardiologists in accordance with EACVI guidelines.

Variables such as TACT and annular valve dimensions were included based on emerging evidence suggesting their relevance in surgical cardiovascular risk prediction, despite being underrepresented in conventional risk models. Their inclusion allowed for a more nuanced analysis of atrial function and electromechanical interactions. More established predictors, such as age, creatinine, ejection fraction, and common comorbidities, were incorporated due to their well-documented prognostic value. This structured approach to variable selection was designed to integrate both traditional and underutilized parameters, enhancing the model’s capacity to capture multifactorial influences on postoperative cardiovascular risk.

### 2.3. Statistical Analysis

All statistical analyses were performed using the IBM SPSS Statistics software, version 26.0 (IBM Corp., Armonk, NY, USA), except for receiver operating characteristic (ROC) curves, which were constructed using Python 3.11 with the scikit-learn library due to its superior support for customized ROC plotting and AUC calculation. Descriptive statistics were calculated for all study variables. Continuous variables were summarized as the mean ± the standard deviation (SD) for normally distributed data or as the median and interquartile range (IQR) for non-normally distributed variables. The Shapiro–Wilk test was used to assess normality. Categorical variables were expressed as absolute frequencies and corresponding percentages. For all statistical analyses, the occurrence of MACE was defined as the positive class (coded as 1), and its absence was defined as the negative class (coded as 0). To assess differences between patients who developed MACE and those who did not, we used the independent samples *t*-test for continuous variables with a normal distribution, and the Mann–Whitney U test was used for non-normally distributed data. The chi-square test or Fisher’s exact test was used to compare categorical variables, depending on expected cell counts. Univariate analysis was conducted to identify potential predictors of MACE. Variables with a *p*-value of <0.05 in univariate testing were considered for further evaluation. In addition to odds ratios and *p*-values, model performance was assessed for each variable individually using logistic regression on a stratified 80:20 train–test split. The classification threshold was set at 0.5, and predictive performance was evaluated using AUC-ROC, accuracy, sensitivity, specificity, precision, and F1-scores. Subsequently, a multivariate logistic regression model was constructed to identify independent predictors of MACE. A backward stepwise approach was applied, and only variables that remained statistically significant (*p* < 0.05) were retained in the final model. Results were presented as regression coefficients (β), odds ratios (ORs), 95% confidence intervals (CIs), and *p*-values. For variables with complete separation (i.e., zero events in the one-outcome group), the odds ratios and confidence intervals were not estimable and are indicated as missing. To evaluate the discriminative power of individual predictors and the final multivariate model, ROC curves were generated using Python. The area under the curve (AUC) was calculated for each variable with univariate significance, as well as for the combined logistic regression model. All tests were two-tailed, and a *p*-value of <0.05 was considered statistically significant throughout the analysis.

### 2.4. Machine Learning Approach

The development and validation of machine learning models in this study were implemented using Python (version 3.10). All analytical procedures, including data preprocessing, model training, and evaluation, were carried out within this environment. Prior to modeling, missing values were imputed using the multivariate imputation by chained equations (MICE) technique. The overall proportion of missing data was low, affecting fewer than 5% of the total dataset. Missing values were observed in three variables: HDL cholesterol, plasma fibrinogen, and TACT, with individual missingness rates ranging from 2.3% to 4.6%. After imputation, continuous variables were standardized using z-score normalization. To prevent data leakage, normalization parameters (mean and standard deviation) were calculated from the training set only and then applied to the test set. Given the moderate class imbalance between MACE and non-MACE patients (55.7% vs. 44.3%), the synthetic minority oversampling technique (SMOTE) was employed to balance class representation. SMOTE was applied exclusively to the training set after the initial 80:20 stratified split, in accordance with best practices to avoid information leakage into the test set. We opted for oversampling rather than undersampling due to the relatively small overall sample size, in order to preserve information from the majority class. While undersampling may reduce class imbalance, it also risks discarding potentially informative samples and decreasing model stability in limited datasets. Feature selection was guided by a combination of clinical plausibility, significance in univariate logistic regression analyses (*p* < 0.05), and redundancy reduction through correlation matrix screening, thereby minimizing multicollinearity. Using the refined feature set, we trained the Extreme Gradient Boosting (XGBoost) model with xgboost library (version 1.7.6), a powerful ensemble learning algorithm capable of capturing non-linear relationships and complex feature interactions. In addition to XGBoost, we evaluated the performance of several other commonly used classifiers, including logistic regression, Random Forest, LightGBM (LightGBM, version 4.2.0), and CatBoost (version 1.2.2). All models were implemented using their respective scikit-learn or native Python libraries and were trained on the same stratified 80:20 train–test split. Hyperparameters for each model were optimized using grid search within 5-fold cross-validation on the training set. Performance metrics including AUC-ROC, accuracy, precision, recall, and F1-scores were calculated on the held-out test set for each model. To further evaluate model generalizability and robustness, we performed 10 independent random 80:20 stratified train–test splits. In each iteration, the XGBoost model was retrained on a new training set and evaluated on the corresponding test set using the same performance metrics. This approach enabled us to assess the stability of model performance across different data partitions. To mitigate the risk of overfitting and assess model robustness, we applied a stratified 5-fold cross-validation step within the training set. To enhance transparency and clinical interpretability of model outputs, SHapley Additive exPlanations (SHAPs) (version 0.41.0) were utilized. SHAP analysis enabled detailed attribution of feature contributions to individual predictions and overall model behavior, revealing both dominant predictors and their directional impact. This methodological choice was crucial to bridge the gap between computational modeling and practical clinical application. The primary goal of machine learning application in this context was not solely predictive performance, but the discovery of clinically meaningful, reproducible predictors of MACE in patients undergoing CABG and/or AVR procedures.

## 3. Results

A total of 131 patients were included in this study, of whom 73 (55.7%) experienced MACE, while 58 (44.3%) did not. The surgical distribution in the study cohort was as follows: A total of 100 patients (76.3%) underwent isolated CABG, while 19 patients (14.5%) underwent isolated AVR, and 12 patients (9.2%) underwent combined CABG and AVR procedures. The baseline characteristics of the two groups are presented in Table 1.

There were no significant differences between the MACE and non-MACE groups regarding gender distribution (*p* = 1.000), presence of diabetes mellitus (*p* = 1.000), hypertension (*p* = 0.7201), previous myocardial infarction (*p* = 0.3143), or other comorbidities including chronic obstructive pulmonary disease (*p* = 0.2502) and peripheral vascular disease (*p* = 0.3335). The intraoperative use of milrinone was rare and not significantly associated with MACE occurrence (*p* = 0.378). However, patients who experienced MACE were significantly older compared to those who did not (67.63 ± 7.16 vs. 63.03 ± 8.60 years, *p* = 0.0011). POAF was also more frequent among the MACE group (32.9% vs. 17.2%, *p* = 0.0461). Among the echocardiographic parameters, TACT was significantly associated with MACE (128.62 ± 21.72 vs. 104.25 ± 11.60 ms, *p* < 0.01), as well as a reduced mitral annular orifice area (4.29 ± 0.33 vs. 4.59 ± 0.23 cm^2^, *p* < 0.01) and a smaller tricuspid annular orifice area (7.52 ± 0.54 vs. 8.10 ± 0.40 cm^2^, *p* < 0.01). Other laboratory and hemodynamic parameters, including BMI, erythrocyte count, creatinine, fibrinogen, HDL cholesterol, cardiac troponin I, and ejection fraction, did not differ significantly between the groups (all *p* > 0.05). The length of stay in the intensive care unit (ICU) was not significantly different between the groups (2.83 ± 2.74 vs. 3.49 ± 19.66 days, *p* = 0.7769).

Univariate logistic regression analysis identified several variables significantly associated with the occurrence of MACE (Table 2). Increasing age was a significant predictor (OR = 1.08, 95% CI: 1.03–1.14, *p* = 0.0015), indicating an 8% increase in odds of MACE per year of age. POAF also significantly increased the risk of MACE (OR = 1.71, 95% CI: 1.21–2.43, *p* = 0.0025), emphasizing its clinical relevance in postoperative management. Among echocardiographic parameters, TACT was strongly associated with MACE (OR = 1.09, 95% CI: 1.05–1.12, *p* < 0.01), suggesting that for every additional millisecond of TACT, the odds of MACE increased by approximately 9%. Moreover, reduced mitral and tricuspid annular orifice areas emerged as protective factors. Specifically, a smaller mitral annular area was associated with markedly lower odds of MACE (OR = 0.03, 95% CI: 0.01–0.13, *p* < 0.01), as was the tricuspid annular orifice area (OR = 0.10, 95% CI: 0.04–0.24, *p* < 0.01), indicating that these structural changes are strong echocardiographic predictors. HDL cholesterol showed borderline significance (OR = 1.69, 95% CI: 1.00–2.87, *p* = 0.0493), suggesting a potentially complex relationship between lipid metabolism and postoperative cardiovascular outcomes. Other clinical, biochemical, and hemodynamic parameters—including BMI, creatinine, erythrocyte count, fibrinogen, troponin I, ejection fraction, and comorbidities (DM, HTA, COPD, previous myocardial infarction)—did not reach statistical significance in the univariate analysis (all *p* > 0.05).

**Figure 1:**
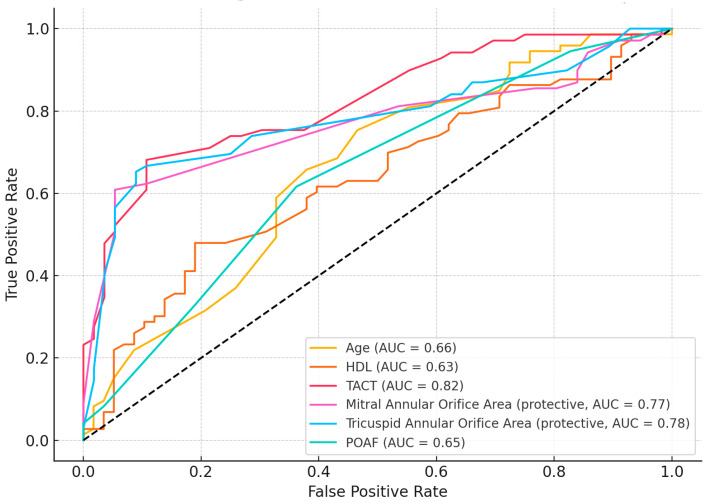
Receiver operating characteristic (ROC) curves for key predictors of MACE.

Receiver operating characteristic (ROC) curve analysis was performed to assess the discriminatory power of individual predictors identified as significant in univariate analysis (Figure 1). Among the evaluated variables, TACT demonstrated the highest predictive accuracy with an area under the curve (AUC) of 0.82, indicating excellent discrimination for MACE occurrence. Two echocardiographic structural parameters also showed strong predictive value: the tricuspid annular orifice area (AUC = 0.78) and the mitral annular orifice area (AUC = 0.77). These two variables were identified as protective factors in logistic regression and showed consistent performance in ROC analysis. Other variables demonstrated moderate discriminative performance: age (AUC = 0.66), POAF (AUC = 0.65), and HDL cholesterol (AUC = 0.63). Despite lower AUC values, these parameters remain clinically relevant and may contribute to risk stratification when combined with more powerful predictors.

**Figure 2:**
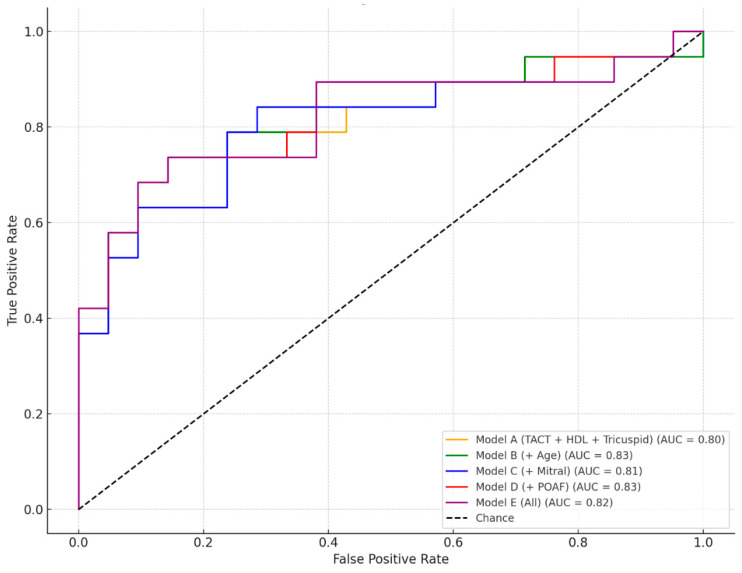
Receiver operating characteristic (ROC) curves of multivariable logistic regression models for prediction of MACE.

We evaluated the discriminative performance of five multivariate logistic regression models incorporating various combinations of clinical and echocardiographic predictors for the occurrence of MACE (Figure 2).

The baseline model, Model A, which included TACT, HDL cholesterol, and the tricuspid annular orifice area, achieved solid predictive accuracy with an AUC of 0.80. This indicates that these three parameters alone provide good discrimination between patients with and without MACE. Building upon this, Model B added age to the predictor set, resulting in the highest observed AUC of 0.83, suggesting that age contributes additional independent value in risk stratification when combined with the core predictors. Model C, which included the mitral annular orifice area instead of age, also maintained strong performance (AUC = 0.81), supporting the relevance of atrioventricular structural markers in predicting MACE. In Model D, POAF replaced age as the fourth variable, yielding a comparable AUC of 0.83, highlighting the predictive importance of rhythm disturbances in the postoperative period. Finally, Model E combined all available predictors (TACT, HDL, tricuspid and mitral annular areas, age, and POAF), but the resulting AUC of 0.82 did not offer a clear advantage over the more parsimonious Models B and D. Taken together, these results underscore that the combination of TACT, HDL, and the tricuspid annular orifice area represents a robust predictive core, and that the addition of either age or POAF further enhances model performance without substantial redundancy. Thus, Model B and Model D emerge as the most effective configurations for MACE prediction in this dataset.

The final multivariate logistic regression model, designated as Model B, included TACT, HDL cholesterol, the tricuspid annular orifice area, and patient age. This configuration yielded the best overall performance, with an area under the ROC curve (AUC) of 0.83, indicating strong discriminative ability for predicting MACE in the postoperative period. Using Youden’s index to define the optimal cut-off point, the threshold for predicted probability was established at 0.64. At this threshold, the model achieved an overall accuracy of 79.4%, with a sensitivity of 69.9% and a specificity of 91.4%. The model’s precision was 91.1%, and the corresponding F1-score was 0.79, indicating a good balance between false positives and false negatives. In terms of individual predictors, TACT emerged as a robust independent risk factor. Each millisecond increase in TACT was associated with a 6% increase in the odds of experiencing MACE (OR = 1.06), with a 95% confidence interval ranging from 1.02 to 1.11 and a *p*-value of 0.002, indicating statistical significance. HDL cholesterol was also positively associated with MACE risk in this cohort, with an odds ratio of 2.04 (95% CI: 1.09–3.83; *p* = 0.027), suggesting that elevated HDL cholesterol in this postoperative context may reflect non-classical risk profiles or inflammatory dysregulation. The tricuspid annular orifice area, in contrast, demonstrated a protective effect; a larger annular area was associated with lower odds of MACE, with an odds ratio of 0.18 (95% CI: 0.05–0.66; *p* = 0.010). Patient age, while included in the model, did not reach statistical significance, with an odds ratio close to unity (OR = 1.02; 95% CI: 0.94–1.11; *p* = 0.611), indicating that its effect was likely attenuated when adjusted for the dominant echocardiographic and biochemical predictors. Overall, this model confirms that a small number of targeted parameters, particularly TACT and tricuspid valve morphology, carry substantial prognostic value. The strong performance of this concise model supports its potential use in future individualized risk stratification strategies following cardiac surgery.

To identify the optimal predictive algorithm for MACE classification, we compared the performance of five machine learning models trained and evaluated on the same stratified 80:20 train–test split. These included XGBoost, Random Forest, LightGBM, CatBoost, and logistic regression. All models were trained using the same input variables and preprocessing pipeline, with SMOTE applied only to the training set and evaluation performed on the held-out test set. Model performance was assessed using five standard classification metrics: AUC-ROC, accuracy, precision, recall, and F1-score. Among the tested models, XGBoost demonstrated perfect classification on the test set, achieving an AUC-ROC of 1.00, an accuracy of 1.00, a precision of 1.00, a recall of 1.00, and an F1-score of 1.00. In contrast, Random Forest, LightGBM, and CatBoost yielded comparable accuracy (0.852) and precision (1.00), but substantially lower recall (0.64) and F1-scores (0.78), indicating poor sensitivity to MACE despite high specificity. Logistic regression achieved the lowest overall performance, with an AUC-ROC of 0.71, an accuracy of 0.63, a precision of 0.53, a recall of 0.73, and an F1-score of 0.62. These findings suggest that XGBoost not only provides superior discrimination but also maintains a balanced sensitivity and specificity profile, which is essential for clinical applicability. Further, the XGBoost model demonstrated consistent performance across 10 independent random 80:20 stratified splits, with a mean AUC of 0.846 ± 0.092, an accuracy of 0.784 ± 0.108, a precision of 0.816 ± 0.097, a recall of 0.800 ± 0.105, and an F1-score of 0.807 ± 0.096. These results indicate stable classification behavior across different data partitions, supporting the robustness and generalizability of the model. Its performance, in conjunction with SHAP-based interpretability, supported its selection as the final model for further analysis. These results indicate that the XGBoost model achieves strong discriminative performance across validation strategies and maintains a favorable trade-off between sensitivity and precision, highlighting its promise as a supportive tool for early identification of high-risk patients. The optimal AUC highlights the model’s robustness in distinguishing between MACE and non-MACE cases across various thresholds.

**Figure 3:**
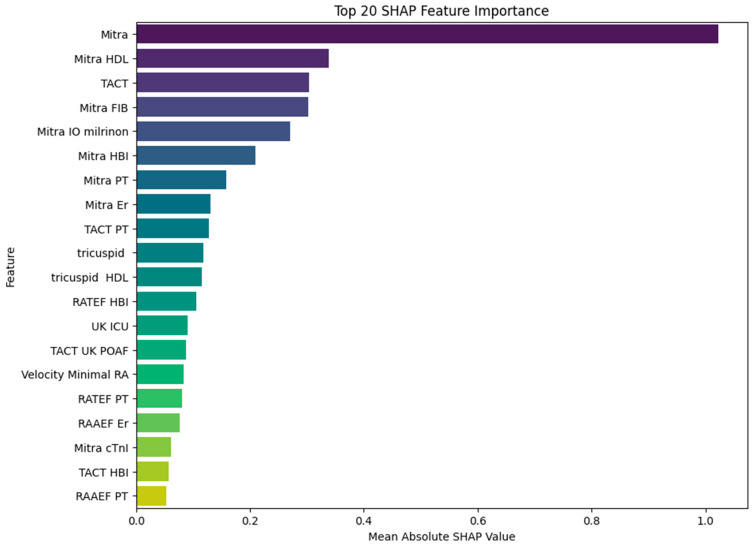
Top 20 predictors ranked by mean absolute SHAP values in the XGBoost model for MACE prediction. The x-axis shows the mean absolute SHAP value, reflecting the average magnitude of each feature’s contribution to the model output. Features are listed on the y-axis in descending order of importance. Higher SHAP values indicate stronger influence on the prediction—regardless of whether the effect is positive or negative. This summary plot provides a global view of feature importance across the entire patient cohort.

To gain deeper insight into the contribution of individual predictors to MACE, we applied SHAP analysis to the trained XGBoost model. The resulting impact of SHAP values on model output is illustrated in Figure 3.

**Figure 4:**
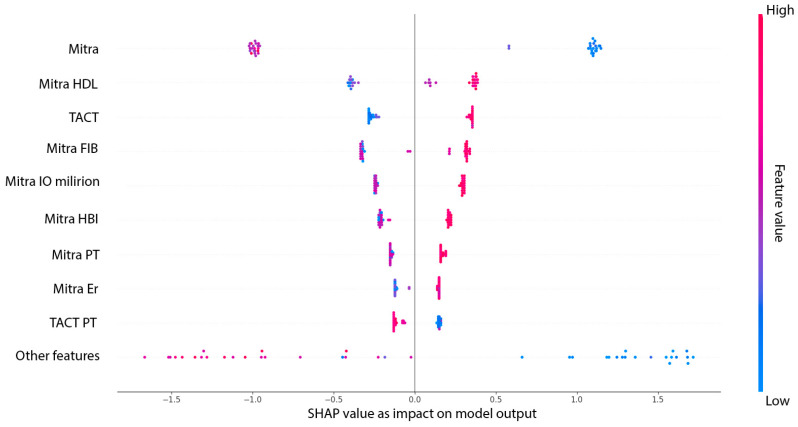
SHAP summary plot (beeswarm) showing the relative importance and direction of individual predictors included in the XGBoost model for MACE classification. Each dot represents a single patient instance, with colors indicating the original feature value (red = high and blue = low). The horizontal position reflects the SHAP value, i.e., the magnitude and direction of the feature’s contribution to the model’s output. Predictors are ranked by the mean absolute SHAP value, with the mitral annular orifice area, HDL cholesterol (in interaction with mitral features), TACT, and fibrinogen emerging as the most influential.

The most impactful variable was the mitral annular orifice area (Mitra), with a mean SHAP value of 1.02, clearly dominating all other predictors. This finding highlights the pivotal role of the mitral valve structure in the pathophysiology of MACE. Interestingly, several of the subsequent top-ranked features reflect interactions between mitral structural alterations and systemic or therapeutic factors, such as HDL cholesterol (SHAP = 0.34), fibrinogen (SHAP = 0.30), and intraoperative milrinone administration (SHAP = 0.27). These results suggest that the prognostic relevance of HDL and fibrinogen may be modulated by the underlying state of the mitral apparatus, and that milrinone—although used selectively—has predictive value primarily in the context of mitral dysfunction. Such variable interactions imply that the model does not rely on isolated predictors but rather captures clinically meaningful combinations of structural, biochemical, and therapeutic dimensions. TACT, which also exhibited strong performance in logistic regression, ranked highly in the SHAP analysis (SHAP = 0.30), further supporting its role as a critical electrophysiological predictor. Additional variables, including intraoperative milrinone administration, hemoglobin, and platelet count, appeared consistently in the top half of contributors, particularly when interacting with mitral or conduction-related domains. The lower-ranked, but still relevant, features included a variety of compound variables involving tricuspid dimensions, right atrial function, and hemodynamic parameters such as minimal right atrial velocity and intensive care unit stay duration. While their individual SHAP values were smaller, they nonetheless contributed to the model’s fine-tuning and improved calibration. In summary, the SHAP analysis reveals that the predictive strength of the XGBoost model stems not only from dominant single predictors such as the mitral valve area or TACT but also from informative combinations of structural and biochemical features—most notably those linking mitral function with HDL and fibrinogen levels. This highlights the importance of integrative physiological interactions in accurately identifying high-risk patients. Figure 4 presents the SHAP summary plot (beeswarm), illustrating both the average impact and the directionality of each predictor’s contribution to the XGBoost model. Each dot represents an individual patient, with colors indicating the relative value of the feature and the position along the X-axis reflecting the magnitude and direction of its influence on model output.

## 4. Discussion

In this study, we identified and validated echocardiographic, biochemical, and clinical predictors of MACE following cardiac surgery, using both conventional statistical methods and machine learning approaches. Our findings highlight the utility of combining structural cardiac parameters with functional and systemic variables to improve postoperative risk stratification.

Univariate and multivariate logistic regression analyses confirmed the prognostic value of TACT, HDL cholesterol, and the tricuspid annular orifice area, with TACT and HDL emerging as independent predictors of MACE. Notably, the protective effect of larger tricuspid annular dimensions suggests that preserved right atrial geometry may mitigate adverse outcomes, possibly by improving preload dynamics and maintaining atrioventricular synchrony. Although age was associated with MACE in univariate analysis, its significance was lost in the multivariate model, suggesting that structural and functional cardiac parameters may subsume the risk typically attributed to chronological age.

In this study, we developed and validated a machine learning (ML) model to predict MACE, CABG, and AVR. Utilizing SHAP analysis, we identified key preoperative and intraoperative variables contributing to postoperative MACE risk, enhancing the interpretability of our predictive model. The XGBoost model further improved predictive performance, achieving an AUC of 1.000 and high accuracy, sensitivity, and precision. Despite the relatively small sample size, the XGBoost model demonstrated stable and reproducible performance across different data partitions. When evaluated over 10 independent random stratified 80:20 splits, classification metrics showed limited variability, with a mean AUC of 0.846 ± 0.092 and an F1-score of 0.807 ± 0.096. This consistency across splits supports the notion that the model was not overly sensitive to a particular training–test configuration and provides additional reassurance regarding its generalizability and resistance to overfitting. In addition to performance evaluation on the independent test set, the XGBoost model was also assessed using 5-fold stratified cross-validation. The average AUC across folds was 0.846 ± 0.082, accuracy was 0.805 ± 0.098, precision was 0.831 ± 0.076, recall was 0.808 ± 0.108, and F1-score was 0.818 ± 0.087, further supporting the stability and robustness of the model across internal partitions. This machine learning approach confirmed that the combination of electrophysiological, biochemical, and morphological parameters yields a robust and well-calibrated classifier for postoperative risk assessments. Importantly, the SHAP analysis revealed that the most influential predictor was the mitral annular orifice area, followed by complex interaction terms combining mitral parameters with systemic markers such as HDL cholesterol, fibrinogen, and milrinone administration. These interactions suggest that the prognostic significance of certain variables is not static, but context-dependent—reflecting the underlying structural integrity of the left atrial–ventricular interface.

Our findings align with previous research demonstrating the superiority of ML models over traditional risk assessment tools. For instance, Shen et al. developed an ML-based model for predicting perioperative MACE in patients with stable coronary artery disease undergoing noncardiac surgery, achieving an area under the receiver operating characteristic curve (AUROC) of 0.898 with the XGBoost algorithm, outperforming the Revised Cardiac Risk Index (RCRI) which had an AUROC of 0.716. Similarly, our model exhibited high predictive accuracy, underscoring the potential of ML approaches in perioperative risk stratification [20].

One potential limitation of the present study is the absence of a direct comparison between our machine learning model and established clinical risk stratification tools such as the EuroSCORE and the Society of Thoracic Surgeons’ (STS) risk calculator. Although these scores are widely used in cardiac surgery, their predictive performance varies across populations and may not fully capture electrophysiological or geometric variables such as TACT and annular valve morphology. Due to incomplete documentation of EuroSCORE and STS parameters in our dataset, we were unable to perform a direct comparison. However, we acknowledge that incorporating such standardized risk metrics in future studies will be essential to assess the added clinical value of data-driven predictive models and to facilitate their integration into surgical decision-making workflows.

SHAP analysis was employed to interpret the relative importance of individual predictors in the XGBoost model. While this method enhances model transparency and may generate hypotheses regarding underlying physiological mechanisms, it should not be interpreted as confirming causal relationships. Moreover, the explanatory value of SHAP is inherently linked to the model’s validity. Given the lack of external validation and the absence of prior feature filtering, the presented SHAP results should be considered exploratory. Their clinical relevance must be confirmed in future studies using larger cohorts and more structured model development pipelines. SHAP analysis revealed that the mitral annular orifice area had the highest mean absolute SHAP value, indicating its significant impact on predicting MACE. This finding aligns with previous studies demonstrating that alterations in mitral annular geometry can substantially affect hemodynamics and clinical outcomes. For instance, Al Amri et al. found that specific mitral annular geometric features influence the feasibility of percutaneous therapy in patients with recurrent mitral regurgitation after surgical annuloplasty [21]. Similarly, Kainuma et al. reported that restrictive mitral annuloplasty leads to sustained improvements in hemodynamics and left ventricular function in patients with functional mitral regurgitation [22]. Furthermore, Kim et al. demonstrated that dilation of the mitral annulus increases the risk of residual or recurrent mitral regurgitation following percutaneous intervention, highlighting the importance of annular geometry in predicting therapeutic response [23].

TACT emerged as a significant predictor in our study. Prolonged TACT has been associated with an increased risk of POAF following CABG, which in turn is linked to higher morbidity and mortality. This finding is consistent with previous research indicating that echocardiographic parameters reflecting atrial conduction, such as the PA-TDI interval, are valuable in predicting POAF. For instance, Özlü et al. demonstrated that prolonged PA-TDI duration is an independent predictor of POAF in patients undergoing CABG [24]. Furthermore, a systematic review and meta-analysis by Kawczynski et al. reinforced the prognostic value of TACT, along with other echocardiographic markers like the left atrial volume index (LAVI) and peak atrial longitudinal strain (PALS), in forecasting POAF across diverse cardiac surgical populations [25]. Our results corroborate these findings, underscoring the importance of atrial conduction parameters in postoperative risk assessments.

Elevated fibrinogen levels were also identified as a key predictor [26]. Fibrinogen, a key glycoprotein involved in coagulation and inflammation, has been independently associated with increased cardiovascular risk. Elevated fibrinogen levels have been linked to higher incidences of postoperative complications, including renal impairment and extended hospital stays. Additionally, MAC has been recognized as an independent risk factor for cardiovascular morbidity and mortality. The concurrent presence of high fibrinogen levels and MAC may reflect a synergistic effect, amplifying the risk of adverse cardiovascular outcomes. This underscores the importance of comprehensive preoperative assessments, integrating both biochemical and echocardiographic parameters, to enhance risk stratification and guide perioperative management strategies [27,28].

Hemoglobin levels, indicative of the patient’s oxygen-carrying capacity, were also significant in our model. Preoperative anemia, defined by reduced hemoglobin concentration, is a well-established independent risk factor for adverse cardiovascular outcomes. Studies have demonstrated that patients with preoperative anemia undergoing cardiac surgery experience higher rates of mortality and postoperative complications, including renal dysfunction, stroke, atrial fibrillation, and prolonged hospital stays [29,30]. For instance, Miceli et al. found that anemic patients had a threefold risk of death and a significantly increased incidence of postoperative renal dysfunction and extended hospitalization [29]. These findings underscore the importance of assessing and optimizing hemoglobin levels preoperatively to mitigate the risk of postoperative complications.

In our study, the tricuspid annular orifice area emerged as a significant predictor of MACE. This finding aligns with recent research highlighting the prognostic value of tricuspid annular geometry in patients with tricuspid regurgitation (TR). For instance, Arfsten et al. demonstrated that tricuspid annular dimensions, rather than traditional measures of TR severity, were significantly associated with all-cause mortality in patients with isolated severe TR [31]. Similarly, Otto et al. found that alterations in tricuspid annular geometry impacted short-term outcomes following percutaneous edge-to-edge repair for severe TR [32]. These studies underscore the importance of assessing TAOA as part of comprehensive risk stratification in patients undergoing cardiac surgery.

Interestingly, HDL cholesterol was associated with increased odds of MACE in univariate logistic regression (OR = 1.69; 95% CI = 1.00–2.87), suggesting a potentially paradoxical pro-risk relationship in this specific cohort. However, SHAP analysis within the multivariate XGBoost model revealed an inverse contribution of HDL to the predicted MACE probability, consistent with its known cardioprotective role in the general population. This apparent contradiction may reflect non-linear interactions or hidden confounding effects captured by the machine learning model but not accounted for in univariate analysis. It also highlights the need for cautious interpretation of variable importance and directionality in complex models, particularly in small datasets. Further studies are needed to clarify the role of HDL in surgical risk prediction and to validate these findings in larger cohorts.

To place these findings into proper context, several limitations must be acknowledged—particularly those arising from the nature of the study design and cohort characteristics. While our sample size was modest, it reflects the complexity and logistical demands of prospective data collection in cardiac surgery settings. The findings presented here should be interpreted as preliminary and hypothesis-generating. Importantly, while internal validation strategies were implemented, external validation on larger and more heterogeneous cohorts will be essential to assess the true clinical utility and generalizability of the proposed predictive model.

While the findings underscore the potential clinical value of the proposed model, several limitations should be acknowledged to contextualize the results and guide future research. The retrospective, single-center design may limit the generalizability of our findings and introduce selection bias. The relatively small number of patients undergoing AVR or combined procedures limited our ability to assess whether the model performs differently across surgical subgroups. Future studies with larger and more balanced cohorts should investigate whether tailored models or interaction terms may improve predictive accuracy in specific surgical populations. The inclusion of POAF in the composite MACE outcome may limit comparability with studies using more conventional definitions focused solely on irreversible events such as myocardial infarction, stroke, or death. Some predictive features may act as surrogates for unmeasured confounders or reflect center-specific practices rather than universally applicable mechanisms. The relatively modest sample size may also affect the robustness of complex feature interactions modeled by XGBoost. Although the XGBoost model achieved perfect classification metrics (AUC = 1.00, sensitivity = 1.0, and precision = 1.0) in the initial train–test split, this finding must be interpreted with caution. Such performance is highly uncommon in clinical prediction settings and likely reflects the influence of data sparsity and potential feature redundancy, particularly in a relatively small dataset. To address this concern, we conducted additional validation through 10 independent stratified random splits and 5-fold cross-validation, both of which demonstrated more realistic performance levels (AUC ≈ 0.846 and F1-score ≈ 0.81), suggesting that the original result may overestimate true generalizability. These findings underscore the importance of cautious interpretation in small-sample machine learning studies and the need for external validation. We also acknowledge that the absence of a formal feature selection strategy may have contributed to overfitting, and future work should aim to incorporate dimensionality reduction or variable filtering to enhance model robustness. One of the key limitations of this study is the absence of a formal feature selection or dimensionality reduction procedure prior to model development. Although all available clinical, biochemical, and echocardiographic variables were included to preserve potential signal in a small dataset, this approach may have introduced noise or redundancy and increased the risk of overfitting. The unusually high performance observed in the initial test split (AUC = 1.00) must therefore be interpreted with caution. While additional analyses across multiple random data partitions and cross-validation demonstrated more realistic performance levels, future studies should incorporate structured predictor filtering or regularization techniques to enhance model robustness and clinical applicability. Additionally, while SHAP enhances model transparency, it does not establish causal relationships. Clinical integration remains theoretical, pending prospective validation and real-world testing.

## 5. Conclusions

This study explores the potential of a machine learning–based model that integrates both conventional and novel echocardiographic and biochemical predictors to improve preoperative risk stratification for MACE in patients undergoing CABG and/or AVR. The inclusion of underutilized but physiologically relevant variables—such as TACT, mitral and tricuspid annular orifice areas, and HDL cholesterol—into the XGBoost algorithm enabled a more comprehensive and individualized assessment of perioperative cardiovascular risk, beyond traditional clinical scoring systems.

Model interpretability was enhanced through SHAP analysis, which provided clinically intuitive insights into the direction and magnitude of each predictor’s contribution to the model’s output. This level of transparency is particularly valuable in high-stakes clinical contexts, where black-box models may face resistance. However, these findings should be interpreted as preliminary. The explanatory power of SHAP is inherently tied to the model’s generalizability, and in the absence of external validation and formal feature selection, the risk of overfitting remains non-negligible. As such, the presented results are best viewed as hypothesis-generating and illustrative of how explainable artificial intelligence (AI) may eventually support decision-making in complex surgical populations.

In conclusion, this study demonstrates the feasibility and clinical promise of applying interpretable machine learning techniques to perioperative cardiovascular risk modeling. By combining advanced analytics with physiologically meaningful variables and transparent outputs, this framework lays the groundwork for individualized, data-informed decision support in high-risk surgical settings. Nonetheless, further research is essential to validate these findings in larger, multicenter cohorts; implement systematic feature selection; and evaluate clinical integration pathways. Only through such rigorous validation can the full potential of interpretable machine learning be realized in precision cardiovascular medicine.

## Figures and Tables

**Table 1:** Baseline demographic, clinical, laboratory, and echocardiographic characteristics in patients with and without MACE.

Variable	MACE = No	MACE = Yes	*p*-Value
Gender	female = 14/58 (24.1%), male = 44/58 (75.9%)	female = 18/73 (24.7%), male = 55/73 (75.3%)	1
DM	yes = 17/58 (29.3%)	yes = 22/73 (30.1%)	1
HTA	yes = 53/58 (91.4%)	yes = 69/73 (94.5%)	0.7201
HBI	yes = 3/58 (5.2%)	yes = 6/73 (8.2%)	0.7361
HOBP	yes = 8/58 (13.8%)	yes = 17/73 (23.3%)	0.2502
PVB	yes = 4/58 (6.9%)	yes = 10/73 (13.7%)	0.3335
IM	yes = 43/58 (74.1%)	yes = 47/73 (64.4%)	0.3143
IO Milrinon	yes = 2/58 (3.4%)	yes = 0/73 (0.0%)	0.378
POAF	yes = 27/58 (46.6%)	yes = 24/73 (32.9%)	0.0461
Age	63.03 ± 8.60	67.63 ± 7.16	0.0011
BMI	28.79 ± 4.88	28.38 ± 4.31	0.6113
Er	4.67 ± 0.49	6.51 ± 16.44	0.3958
Creat	94.09 ± 22.06	94.42 ± 20.95	0.9292
FIB	4.49 ± 0.78	4.50 ± 0.95	0.9632
HDL	1.31 ± 0.73	3.55 ± 16.45	0.3034
cTnI	0.14 ± 0.87	0.12 ± 0.72	0.8838
EF	49.47 ± 9.73	49.58 ± 10.80	0.952
TACT	104.25 ± 11.60	128.62 ± 21.72	<0.01
Mitral Annular Orifice Area	4.59 ± 0.23	4.29 ± 0.33	<0.01
Tricuspid Annular Orifice Area	8.10 ± 0.40	7.52 ± 0.54	<0.01
EKK Min	98.69 ± 91.81	113.84 ± 62.73	0.2652
UK ICU	3.49 ± 19.66	2.83 ± 2.74	0.7769

DM—Diabetes Mellitus, HTA—Hypertension Arterialis, HBI—Hemoglobin, HOBP—Chronic Obstructive Pulmonary Disease (COPD), PVB—Peripheral Vascular Disease, IM—Myocardial Infarction, IO Milrinon—Intraoperative Use of Milrinone, POAF—Postoperative Atrial Fibrillation, Age—Patient Age, BMI—Body Mass Index, Er—Erythrocyte Count, Creat—Serum Creatinine, FIB—Fibrinogen, HDL—High-Density Lipoprotein Cholesterol, cTnI—Cardiac Troponin I, EF—Left Ventricular Ejection Fraction, TACT—Total Atrial Conduction Time, Mitral Annular Orifice Area—Area of the Mitral Valve Annulus, Tricuspid Annular Orifice Area—Area of the Tricuspid Valve Annulus, EKK Min—Minimal Velocity of Early Diastolic Filling (Echo-Derived), and UK ICU—Length of Stay in the Intensive Care Unit.

**Table 2:** Univariate logistic regression analysis of predictors associated with MACE.

Variable	OR	95% CI	AUC	Accuracy	Sensitivity	Specificity	Precision	F1-Score	*p*-Value
Age	1.08	1.03–1.14	0.644	0.704	0.933	0.417	0.667	0.778	0.0015
BMI	0.99	0.91–1.06	0.594	0.556	1	0	0.556	0.714	0.7233
Creat	1	0.99–1.02	0.542	0.556	1	0	0.556	0.714	0.6541
cTnI	0.96	0.62–1.49	0.475	0.519	0.933	0	0.538	0.683	0.8661
DM	1.07	0.50–2.30	0.467	0.556	1	0	0.556	0.714	0.8546
EF	1	0.97–1.04	0.464	0.556	1	0	0.556	0.714	0.9511
EKK Min	1	1.00–1.01	0.536	0.444	0.8	0	0.5	0.615	0.2309
Er	1.03	0.93–1.14	0.433	0.556	1	0	0.556	0.714	0.6073
FIB	0.96	0.65–1.43	0.461	0.556	1	0	0.556	0.714	0.8543
Gender	1.1	0.48–2.52	0.475	0.556	1	0	0.556	0.714	0.8136
HBI	2.11	0.39–11.31	0.483	0.556	1	0	0.556	0.714	0.3837
HDL	1.69	1.00–2.87	0.619	0.556	1	0	0.556	0.714	0.0493
HOBP	1.81	0.71–4.61	0.5	0.556	1	0	0.556	0.714	0.2127
HTA	1.59	0.41–6.24	0.417	0.556	1	0	0.556	0.714	0.5037
IM	0.63	0.29–1.37	0.617	0.556	1	0	0.556	0.714	0.2387
IO Milrinon	–	–	0.5	0.556	1	0	0.556	0.714	0.9995
Mitral	0.03	0.01–0.13	0.864	0.72	0.857	0.545	0.706	0.774	<0.010
POAF	1.71	1.21–2.43	0.594	0.519	0.6	0.417	0.562	0.581	0.0025
PVB	2.2	0.65–7.45	0.458	0.556	1	0	0.556	0.714	0.2036
TACT	1.09	1.05–1.12	0.987	0.92	0.857	1	1	0.923	<0.010
Tricuspid	0.1	0.04–0.24	0.857	0.8	0.786	0.818	0.846	0.815	<0.010
UK ICU	1	0.97–1.02	0.622	0.593	0.6	0.583	0.643	0.621	0.7565

Age—Patient Age, BMI—Body Mass Index, Creat—Serum Creatinine, cTnI—Cardiac Troponin I, DM—Diabetes Mellitus, EF—Left Ventricular Ejection Fraction, EKK Min—Minimal Velocity of Early Diastolic Filling (Echo-Derived), Er—Erythrocyte Count, FIB—Fibrinogen, HBI—Hemoglobin, HDL—High-Density Lipoprotein Cholesterol, HOBP—Chronic Obstructive Pulmonary Disease (COPD), HTA—Hypertension Arterialis, IM—Myocardial Infarction, IO Milrinon—Intraoperative Use of Milrinone, Mitral Annular Orifice Area (Mitral)—Area of the Mitral Valve Annulus, POAF—Postoperative Atrial Fibrillation, PVB—Peripheral Vascular Disease, TACT—Total Atrial Conduction Time, Tricuspid Annular Orifice Area (Tricuspid)—Area of the Tricuspid Valve Annulus, UK ICU—Length of Stay in the Intensive Care Unit; “–” indicates that the odds ratio and confidence interval could not be estimated due to complete separation (i.e., no events occurred in one subgroup).

## Data Availability

The data that support the findings of this study are available upon request from the corresponding author.
